# A cross-sectional study comparing lateral and diagonal maximum weight shift in people with stroke and healthy controls and the correlation with balance, gait and fear of falling

**DOI:** 10.1371/journal.pone.0183020

**Published:** 2017-08-15

**Authors:** Margaretha M. van Dijk, Sarah Meyer, Solveig Sandstad, Evelyne Wiskerke, Rhea Thuwis, Chesny Vandekerckhove, Charlotte Myny, Nitesh Ghosh, Hilde Beyens, Eddy Dejaeger, Geert Verheyden

**Affiliations:** 1 UZ Leuven — University Hospitals Leuven, Department of Physical Medicine and Rehabilitation, Pellenberg, Belgium; 2 KU Leuven – University of Leuven, Department of Rehabilitation Sciences, Leuven, Belgium; 3 UZ Leuven — University Hospitals Leuven, Department of Geriatrics, Pellenberg, Belgium; Northwestern University, UNITED STATES

## Abstract

Impaired balance is common post stroke and can be assessed by means of force-platforms measuring center of pressure (COP) displacements during static standing, or more dynamically during lateral maximum weight shift (MWS). However, activities of daily life also include diagonal MWS and since force platforms are nowadays commercially available, investigating lateral and diagonal MWS in a clinical setting might be feasible and clinically relevant. We investigated lateral and diagonal MWS while standing in patients with stroke (PwS) and healthy controls (HC), evaluated MWS towards the affected and the non-affected side for PwS and correlated MWS with measures of balance, gait and fear of falling. In a cross-sectional observational study including 36 ambulatory sub-acute inpatients and 32 age-matched HC, a force platform (BioRescue, RM Ingénierie, France) was used to measure lateral and diagonal MWS in standing. Clinical outcome measures collected were Berg Balance Scale and Community Balance and Mobility Scale (CBMS) for balance, 10-meter walk test (10MWT) for gait speed and Falls Efficacy Scale–international version for fear of falling. MWS for PwS towards the affected side was significantly smaller compared to HC (lateral: p = 0.029; diagonal-forward: p = 0.000). MWS for PwS was also significantly reduced towards the affected side in the diagonal-forward direction (p = 0.019) compared to the non-affected side of PwS. Strong correlations were found for MWS for PwS in the diagonal-forward direction towards the affected side, and clinical measures of balance (CBMS: r = 0.66) and gait speed (10MWT: r = 0.66). Our study showed that ambulatory sub-acute PwS, in comparison to HC, have decreased ability to shift their body weight diagonally forward in standing towards their affected side. This reduced ability is strongly related to clinical measures of balance and gait speed. Our results suggest that MWS in a diagonal-forward direction should receive attention in rehabilitation of ambulatory sub-acute PwS in an inpatient setting.

## Introduction

Stroke is one of the leading causes of disability in adulthood in the developed world, and has a wide range of clinical manifestations [1,2]. Due to a combination of motor, sensory and cognitive impairments, a high proportion of patients with stroke (PwS) experience balance deficits and subsequently, restricted activities of daily living [3–6]. Tyson et al. [3] reported that 83% of PwS experience balance impairments, typically observed as asymmetry in weight bearing towards the unaffected side. Other studies confirmed this concept of a shifted center of pressure (COP) towards the unaffected side [7–9]. Furthermore, an increased postural sway is often observed, which along impaired balance and decreased weight shifting abilities, are considered to be contributing factors for falls in PwS [10–12].

Force platforms are frequently used to quantitatively measure COP in standing and thereby interpret standing stability or postural control strategies [9,13–19]. Previous studies investigating stance symmetry and standing balance focused on measuring anterior-posterior (AP) and/or lateral (L) excursion of COP [13–16]. Roerdink et al. [17] reported that the range of COP excursion and velocity was greater towards the non-affected side compared to the affected side during three quiet standing conditions: eyes open, eyes open while performing a dual task and eyes closed. Similar findings were reported by Eng and Chu investigating chronic PwS [18]. They found that the weight-bearing ability was better towards the non-affected side and that COP excursions were smaller towards the affected side, especially during forward weight shifting. De Haart and colleagues investigated the restoration of weight shifting capacity after stroke and found that even subjects with severe stroke are able to improve their speed and precision of weight shifting [19].

Balance, gait velocity and fear of falling are commonly assessed in PwS to evaluate progress. In the clinical setting, different standardized scales are used to assess balance [20,21]. Studies have correlated AP and L COP displacements in quiet standing to various standardized balance scales. Significant relations have been reported between altered static COP excursion and velocity in the AP or L direction and lower scores on balance outcomes [14–16,22]. Previous studies have also found that COP excursion in the L direction is a predictor of gait velocity. Nardone et al. showed that increased asymmetrical weight bearing in the L direction in standing was significantly correlated with lower gait velocity [8]. Mizelle and colleagues reported that PwS with greater L COP displacement during walking showed higher gait velocity [23]. Force platform variables have also been correlated with risk of falls. A review of Piirtola and Era suggests that force platform data provides predictive value for subsequent falls [24]. Although different force platform variables were previously shown to be associated with the risk of falls in older people, up to now it remains unknown whether these variables are also related to fear of falling in PwS.

COP variables collected in the above-mentioned studies were obtained during either quiet standing or gait but did not investigate voluntary maximum weight shift (MWS) which could provide a dynamic component of balance ability. Another element of the aforementioned studies is the use of the AP and/or L directions when assessing stance symmetry and balance. Lateral weight shift is a key component in treatment. For instance, it is common practice during gait training to assist PwS in lateral weight shift, in order to bear weight on one leg and bring the other leg forward. However, when performing activities of daily life, weight shifting is not restricted to either the AP or L direction, but is conducted in diagonal directions as well. Up to 95% of activities of daily life (ADL) involve both arm and trunk movement and therefore, comprise diagonal weight shifting during reaching, transfer or turning activities [25]. Reaching or leaning are potential situations in which a fall can occur [26], and together with the fact that weight shifting capacity can be improved post stroke [19], it seems important to investigate diagonal MWS in PwS. Recent literature shows that traditional Chinese exercise like Tai Chi improves balance [27]. One of the aspects that could account for this improvement is the constant shifting of the body weight over a stable base of support in all directions, including diagonally. A study by Han et al showed that the center of gravity path during level walking has a similar shape as the tilde symbol (~) and that the COP coordinates during stance phase are oriented 6° inwards [28]. This would suggest a relation between diagonal weight shift and gait. Previous studies did measured MWS in the diagonal direction but in different populations; traumatic brain injury, Parkinson’s disease and progressive supra-nuclear palsy [29–31]. To the best of our knowledge, there is no literature that evaluated MWS in the diagonal direction in PwS.

In order to know if diagonal weight shift should also be evaluated in PwS, or considered as part of treatment, we should first investigate if diagonal weight shifting ability is altered in PwS and if there is a relation with clinical outcome measures. Nowadays there are more and more affordable, easy to use force platforms available, which provide clinicians with the opportunity to integrate them into clinical practice. In combination with real-time visual feedback, some systems allow assessment of MWS without reaching with the arms and thus allow inclusion of people with a hemiparetic or hemiplegic arm. Therefore, the present study investigated MWS in PwS and their relation with clinical measures of balance, gait and fear of falling. The objective of this study is three-fold: first, to compare lateral and diagonal MWS in PwS and healthy controls (HC). Secondly, to compare lateral and diagonal MWS towards the affected and non-affected in PwS. And thirdly, to determine the correlations between MWS in PwS and clinical outcome measures for balance, gait and fear of falling. Based on previous literature, we hypothesized a reduced diagonal MWS for PwS in comparison with healthy controls. Furthermore, we hypothesized decreased diagonal MWS towards the affected side compared to the non-affected side in PwS, and that diagonal MWS have a significant moderate to strong correlation with clinical outcome measures. Should our hypotheses be confirmed, assessing diagonal MWS could become integrated in the assessment of PwS and eventually part of their rehabilitation program.

## Methods

### Participants and setting

The present study was conducted between March 2013 and March 2016 and had a cross-sectional observational design. A total of 36 PwS were recruited for this study, all were admitted to the University Hospitals Leuven, campus Pellenberg (Belgium), at the non-acquired brain injury and geriatric rehabilitation ward. Inclusion criteria were 1) diagnosed with a first-ever stroke, 2) admitted to the inpatient rehabilitation centre, 3) being able to stand independently for more than two minutes, and 4) signed informed consent. Exclusion criteria were 1) a musculoskeletal or other neurological disorder that would interfere with the study protocol, and 2) communication or speech problems that would interfere with the testing procedure. A total of 32 age-matched healthy elderly were assessed as healthy controls (HC). Inclusion criteria were 1) age above 50, 2) being able to stand independently for more than two minutes, and 3) signed informed consent. The exclusion criteria were 1) conditions that could negatively influence balance, and 2) a Berg balance scale score below 54.

Ethical approval for this study and for the written informed consent was obtained from the Medical Ethics Committee of the University Hospitals Leuven, S55261 and S57757.

### Test protocol

In order to investigate MWS in PwS and HC, we used a commercially available force platform (BioRescue, RM Ingénierie, France). To correlate MWS with balance, gait and fear of falling, we included a total of four clinical outcome measures; Berg balance scale (BBS) and community balance and mobility scale (CBMS) to measure balance, 10-meter walk test (10MWT) for assessing gait speed and falls efficacy scale—international version (FES) for evaluating fear of falling.

### Maximum weight shift: Force platform protocol

Our force platform (BioRescue, RM Ingénierie, France) allows assessment of MWS by measuring the displacement of the COP during the MWS in the anteroposterior (AP), lateral (L), diagonal-forward and diagonal-backward direction. The force platform (610×580×10 mm) consists of 1600 separate resistive sensors, all of which have a fixed place in a grid, and uses a 30Hz sampling frequency. The corresponding software (Sycomore), calculates the position and displacement of the centre of pressure (COP) and provides instructions and real-time visual feedback on a screen.

Force platforms are considered reliable for measuring balance in standing post stroke [32]. When measuring MWS with the force platform used in the present study, Geronimo et al. found good to high intraclass correlation coefficients (ICC = 0.83–0.95) for intrasession agreement and good coefficients (ICC = 0.78–0.83) for intersession agreement [33].

In this study, we used the “limits of stability, free” modus, which is an evaluation of limits of stability (MWS) while maintaining balance. Participants stood independently with the feet placed on the platform, hip-width apart and in a position in which the participant felt comfortable. Tape was applied on the platform (medial, lateral, anterior and posterior position of each foot) to insure the same position was used for each trial. Visual instruction and feedback was shown on a large TV screen 2m in front of the force platform at eye level. In the middle of the screen was a square with a red dot inside the square representing the participant. When the participant weight shifted, the red dot moved accordingly in real-time. Instructions for the MWS was given visually by an arrow on the screen and verbally by the assessor: “move your weight in the direction of the arrow as far as possible without losing your balance or move your feet and stay there until the arrow disappears.” The trial started when the first arrow appeared on the screen. After eight seconds, the arrow disappeared and the participant was asked to return to the starting position. The next arrow appeared only when the participant was back in the starting position. Eight arrows constituted one trial and appeared randomly in the following directions: North, South, East, West, North East, North West, South East and South West.

Participants conducted five consecutive trials (rest was allowed between trials), whereby the first two trials were used to acquaint the participants to the device and procedures. We used the mean of the finial three trials in our analysis. In a systematic review by Ruhe et al., it is stated that COP variables are reliable when taking an average of between two and seven trials [34].

From the MWS on the force platform the following COP variables were collected: distance (cm) and length (cm) of the lateral (East and West) and diagonal (North East, North West, South East and South West) directions. Distance is the straight line of the COP displacement in one specific direction. Length is the total excursion of the COP displacement, from the starting position to the furthest point in one direction.

A standardized protocol was applied stating how the test was explained to the participants, the exact wording that was used during the assessment and where the evaluators were standing to provide safety if necessary. Two assessors were present for the duration of the protocol; one assessor stood next to the PwS on their affected side to provide safety and the other assessor controlled the device and gave verbal instructions. The protocol was standardized across evaluators trough piloting, supervised by the first author (MVD).

### Clinical outcome measures

In order to assess balance we chose two measurements; the BBS because this is probably the most commonly used balance scale for PwS in research and clinical practise, and the CBMS, since we wanted to include a scale that would evaluate more advanced balance skills than the BBS. To assess gait speed, we chose the easy to use and standardized 10MWT and the FES questionnaire was used to evaluate fear of falling, which is a self-reported feasible measure for this aspect.

The BBS is a widely accepted measure of basic balance in PwS with an excellent interrater reliability [20]. It consists of 14 items, which are scored on a 5-point ordinal scale (0–4), and has a maximum score of 56, with a higher score indicating better performance. The CBMS has shown to be a reliable and valid tool for assessing community balance and mobility in ambulatory stroke survivors [35]. Thirteen tasks, like tandem walking, pivot turn and hopping, are scored on a 6-point scale (0–5). The maximum attainable score is 96; a higher score reflects a better performance.

The 10MWT measures the gait velocity in meters per second over a distance of 10m, whereby the mean velocity at comfortable speed is calculated after three trials. Geroin et al. reported that the 10MWT is a highly responsive measurement in PwS [36].

The FES is a reliable and valid questionnaire for measuring fear of falling in PwS [37]. The tool scores the amount of concern for falling while considering 16 different activities of daily living, scoring from 1 to 4 points (maximum 64), where 1 equals no concern at all and 4 equals very concerned of falling during that activity. Therefore, a higher score reflects more fear of falling.

All the outcome measures were performed according to their standardized published guidelines and were part of the standardization and piloting protocol. To minimize fatigue during testing, we paired the tests into three groups: 1) BBS (in addition to collecting patient characteristics); 2) CBMS and 10MWT; and 3) force platform and FES. The pairing of the test was based on time; being able to complete the tests during a one-hour session. The total set of assessments was conducted during three consecutive weekdays. The order of pairs in which the tests were carried out was randomized for each participant.

All collected data can be found in the supporting information S1 dataset.

### Data analysis

Distance and length of the maximum voluntary COP displacement on both sides in the diagonal-forward, the diagonal-backward and lateral direction were collected from the force platform program and categorized into affected and non-affected side for each PwS. For HC, a weight-distribution print was made in the static standing position, one second before the first trial started, to determine the preferred leg of support (PLOS). COP variables towards the PLOS of HC was compared with COP variables towards the non-affected side of PwS.

Descriptive analyses were performed of the COP and clinical variables, and showed that our data was not normally distributed. To compare COP variables of PwS and HC we used Mann Whitney U tests. Non-parametric paired analyses (Wilcoxon signed rank test) were used to determine whether distance and length towards the affected and non-affected side of PwS were significantly different.

For PwS, force platform variables and clinical outcome measures were correlated by using Spearman rho correlation coefficients (r). A strong correlation was defined as r = 0.60–0.80, a moderate correlation as r = 0.40–0.60 and r<0.40 was defined as a low correlation [38]. Level of significance was set at p<0.05. All analyses were conducted with SPSS version 21 (IBM, USA).

## Results

### Participant characteristics

A total of 36 PwS were included, of which eight (22%) were females. Thirteen (36%) individuals had left-sided hemiplegia and 23 (64%) right-sided hemiplegia. Twenty-eight (78%) patients had an ischemic stroke and all participants were ambulatory (Functional Ambulation Category 3 or higher). The mean age (±SD) of PwS was 62 (±14) years with the youngest participant being 22 and the oldest 84. Average time since onset of stroke was 85 (±51) days. We also included 32 healthy elderly (HC), of which 18 (56%) were female and who had a mean age of 65 (±9) years. Thirty (94%) participants preferred their left leg and two (6%) their right leg as PLOS. Descriptive data for both groups can be found in Table 1.

**Table 1:** Participant characteristics.

**Characteristic**	**Healthy controls (n = 32)**				
**Mean age in years (SD)**	64.56 (9.36)				
**Gender: male/female n (%)**	14/18 (44/56)				
**Preferred leg of support: left/right n (%)**	30/2 (94/6)				
	**Patients with stroke (n = 36)**				
**Mean age in years (SD)**	62.19 (14.35)				
**Gender: male/female n (%)**	28/8 (78/22)				
**Affected side: left/right n (%)**	13/23 (36/64)				
**Ischemic/hemorrhagic stroke n (%)**	28/8 (78/22)				
**Mean time since onset of stroke in days (SD)**	84.77 (51.2)				
**Clinical outcome measures**	**median**	**Q1**	**Q3**	**min**	**max**
**BBS**	49	42	56	27	56
**CBMS**	31	15	66	0	90
**10 MWT**	0.93	0.62	1.25	0.16	1.76
**FES**	27	21	32	16	46

BBS = Berg balance scale; CBMS = community balance and mobility scale; 10MWT = 10 meter walking test; FES = falls efficacy scale–international version.

### Force platform variables of MWS: Patients with stroke versus healthy controls

Table 2 shows the comparison of force platform variables between PwS and HC. When comparing the affected side of the PwS with the non-preferred side of support of the HC, two significant differences in COP distance displacement were found. The maximum voluntary weight shift was significantly smaller for the stroke group in the L (p = 0.029) and diagonal-forward direction (p = 0.000), but not in the diagonal-backward direction. No significant differences were found for COP length displacement. Additionally, two significant differences in COP variables were found when comparing the non-affected side of the PwS with the preferred side of support of the HC. COP length displacement was significantly larger for PwS in the L (p = 0.034) and the diagonal-forward direction (p = 0.028) compared to HC. No significant difference was found for COP length displacement in the diagonal-backward direction or for COP distance displacement in any direction.

**Table 2:** Comparison between patients with stroke (n = 36) and healthy controls (n = 32) for force platform parameters.

	**Affected side**			**NPLOS**			**P value**
**Distance (cm)**	Median	Q1	Q3	Median	Q1	Q3	
Lateral	5,1	3,2	6,4	6,1	4,6	7,6	0,029*
Diagonal-forward	4,6	2,7	6,1	6,9	4,7	7,6	0,000*
Diagonal-backward	3,5	2,3	5,3	4,4	3,1	6,3	0,079
**Length (cm)**							
Lateral	12,0	8,4	16,4	11,2	9,0	12,5	0,296
Diagonal forward	11,0	9,0	15,8	11,2	9,0	13,1	0,519
Diagonal-backward	11,9	8,8	17,8	11,9	8,8	13,8	0,499
	**Non-affected side**			**PLOS**			**P value**
**Distance (cm)**	Median	Q1	Q3	Median	Q1	Q3	
Lateral	5,4	3,2	7,1	5,8	4,6	7,6	0,152
Diagonal-forward	5,3	3,2	6,8	6,3	5,0	7,1	0,054
Diagonal-backward	3,6	2,0	5,1	4,3	2,6	6,0	0,090
**Length (cm)**							
Lateral	11,6	8,5	16,4	9,5	7,7	12,2	0,034*
Diagonal-forward	12,6	9,3	17,7	10,9	9,0	11,5	0,028*
Diagonal-backward	11,3	9,4	14,7	11,8	9,6	14,8	0,927

Comparison between patients with stroke and healthy controls for COP distance and COP length in three directions: lateral, diagonal-forward and diagonal-backward, using the Mann Whitney U Test. The affected side of the PwS was compared with the non-preferred leg of support (NPLOS) of the HC and the non-affected side with the preferred leg of support (PLOS).

* p<0,05

### Force platform variables of MWS: Affected versus non-affected side in patients with stroke

When comparing COP variables between affected and non-affected side for PwS, a significant difference for COP distance displacement in the diagonal-forward direction (p = 0.019) was found, with MWS being larger towards the non-affected side. No other significant differences were found (Table 3).

**Table 3:** Comparison between affected and non-affected side for force platform parameters.

	Affected side			Non-affected side			P value
Distance (cm)	Median	Q1	Q3	Median	Q1	Q3	
Lateral	5,1	3,2	6,4	5,4	3,2	7,1	0,272
Diagonal-forward	4,6	2,7	6,1	5,3	3,2	6,8	0,019*
Diagonal-backward	3,5	2,3	5,3	3,6	2,0	5,1	0,567
**Length (cm)**							
Lateral	12,0	8,4	16,4	11,6	8,5	16,4	0,981
Diagonal-forward	11,0	9,0	15,8	12,6	9,3	17,7	0,793
Diagonal-backward	11,9	8,8	17,8	11,3	9,4	14,7	0,383

Comparison between affected and non-affected side for force platform parameters (COP distance and COP length) in three directions: lateral, diagonal-forward and diagonal-backward, using the Wilcoxon Sign rank test (n = 36).

* p<0,05

### Correlations between force platform variables of MWS and clinical outcome measures in patients with stroke

Table 4 shows the correlations between force platform variables and the clinical outcome measures. Moderate to strong positive correlations were found between the CBMS and COP distance displacement in all directions, towards the affected and non-affected side (r = 0.47 to 0.66), indicating a better advanced balance score being related to further MWS. The strongest correlation was found in the diagonal-forward direction towards the affected side. Overall moderate correlations were found for the BBS and COP distance displacement in all directions towards the affected side and non-affected side (r = 0.33 to 0.55), indicating a better basic balance score being related to further MWS. Again, the strongest correlation was found in the diagonal-forward direction towards the affected side. Higher gait speed was moderately to strongly correlated with further COP distance displacement in all directions (r = 0.43 to 0.66), with once again the strongest correlation found in the diagonal-forward direction towards the affected side. Increased fear of falling was associated with lower COP distance displacements in all directions, but correlations were mostly low with only two moderate associations (lateral displacement to affected side r = -0.45, diagonal-forward to non-affected side r = -0.41).

**Table 4:** Spearman’s rho correlation coefficients of the force platform parameters and clinical outcome measures.

	**Affected side**			
**Distance (cm)**	CBMS	BBS	10MWT	FES
Lateral	0,603**	0,505*	0,559*	-0,448*
Diagonal-forward	0,664**	0,550*	0,659**	-0,313
Diagonal-backward	0,471*	0,328	0,429*	-0,286
**Length (cm)**				
Lateral	0,223	0,011	0,290	-0,040
Diagonal-forward	0,198	-0,009	0,240	0,140
Diagonal-backward	0,191	-0,030	0,187	-0,196
	**Non-affected side**			
**Distance (cm)**	CBMS	BBS	10MWT	FES
Lateral	0,613**	0,435*	0,556*	-0,339
Diagonal-forward	0,564*	0,430*	0,523*	-0,410*
Diagonal-backward	0,592*	0,460*	0,575*	-0,376
**Length (cm)**				
Lateral	0,240	0,050	0,289	-0,057
Diagonal-forward	0,251	0,040	0,307	-0,279
Diagonal-backward	0,179	-0,023	0,084	0,014

Spearman’s rho correlation coefficients of the force platform parameters (COP distance, COP length) in three directions; lateral, diagonal-forward and diagonal-backward for both affected and non-affected side with clinical outcome measures: Berg balance scale (BBS), community balance and mobility scale (CBMS), 10-meter walk test (10MWT) and falls efficacy scale–international version (FES) (n = 36).

*indicates moderate correlation (r = 0.41–0.60)

** indicates strong correlation (r = 0.61–0.80)

Only low, non-significant correlations (r = -0.28 to 0.29) between our clinical outcome measures and COP length displacements were found.

## Discussion

The principal findings of this study were in line with our hypotheses and are: (i) PwS showed reduced ability to MWS in the lateral and diagonal-forward direction towards their affected side and an increased instability in MWS towards their non-affected side compared to HC; (ii) in PwS, MWS is significantly smaller in the diagonal-forward direction towards the affected side compared with the non-affected side; and (iii) MWS showed mostly moderate to strong correlations with the outcome measures for balance and gait, with the two strongest correlations in the diagonal-forward direction for the CBMS and 10MWT.

(i) This study showed that PwS have an altered MWS in standing in the lateral and diagonal-forward direction compared to HC. Towards the affected side, PwS showed a significant smaller COP distance while the total trajectory (length) of the COP was comparable to HC. Thus, PwS had a similar trajectory length as the control group, but the actual distance (i.e. straight line towards the furthest point) was significantly smaller compared with HC. On the contrary, towards the non-affected side it was COP length that was significantly larger in PwS, compared to HC with COP distance being similar. Thus, PwS could shift their weight towards their non-affected side comparably as far as the healthy control group towards their preferred side (actual distance), but it took PwS a longer route to get to this point. Together, these results could be explained by PwS having reduced dynamic ability for MWS compared to HC. This is supported by Goldie et al. who stated that PwS have less propulsive force to initiate a COP displacement and provide less appropriate deceleration activity after displacement [39]. Patients would lack coordination between the stabilisation muscles of the pelvis and are unable to generate adequate muscle activity [39].

(ii) Furthermore, when comparing MWS towards affected and non-affected side in PwS, we found that COP distance was significant smaller towards the affected side in the diagonal-forward direction. This might indicate that PwS have a reduced ability to MWS in the diagonal-forward direction towards their affected side. Our results are in line with findings of Eng and Chu who concluded that the forward weight shifting ability in one-foot-forward-standing was lower on the paretic side [18]. No significant differences were found in our study for the lateral COP variables, which is as reported by Goldie et al. [40] and Eng and Chu [18]. A weight bearing asymmetry in standing with a shift towards the non-affected side is common in people post stroke [7–9], and the improved COP stability of the lateral component after rehabilitation as in the study of Paillex and So [41], could account for non-significant findings in our study for lateral COP displacement.

In addition, no significant differences were found for COP variables in the diagonal-backward direction between PwS and HC, or in PwS between affected and non-affected side. Goldie et al. suggested that the relatively short moment arm between the ankle joint and the heel of the foot over which the displacement occurs, limits the backward movement in both legs and could explain this non-significant difference [40]. The moderate to high recovery level of our PwS and the fact that backward displacements are smaller than lateral and diagonal-forward displacements, might further explain our findings.

(iii) Finally, we found moderate to strong correlations for balance (CBMS and BBS) and gait (10MWT) with COP distance, with the strongest correlations in the diagonal-forward direction towards the affected side. These strong correlations and the significant difference for COP distance in PwS, suggest that the ability to MWS in the diagonal-forward direction is of importance, especially for community balance and gait velocity in ambulatory, sub-acute PwS. The strong correlation with the CBMS could be explained by the dynamic items of the scale which challenge balance and gait [35], including tests like tandem walking, forward-turn-backward walking, hopping and running and further high-level tasks which are relevant in daily life and integrate diagonal-forward displacements. The forward progression model of the COP trajectory during level walking by Han et al. further supports our findings [28].

When correlating balance outcome measures with COP variables of MWS, we found that the CBMS showed overall stronger correlations then the BBS. Since moderate correlations were found with the BBS, a certain range of MWS in all directions seems to be needed for basic or static balance. When performing more dynamic and advanced balance activities the range of MWS needed seems to further increase. In our findings, the CBMS seems to be a more suitable outcome measure for ambulatory, sub-acute PwS with moderate to mild mobility impairments, which is in line with findings by Knorr and colleagues [35]. They reported a stronger association between lower limb strength and the CBMS as compared to the BBS.

The present study found moderate correlations with gait speed and COP distance in all directions with one strong correlation (diagonal-forward to the affected side), meaning that the further PwS can weight shift in the diagonal-forward direction, the faster they can walk. Similar positive correlations were also found by Mizelle et al. who utilized pressure sensitive insoles while participants stepped over an instrumented mat allowing for the simultaneous measurement of COP displacements and gait velocity [23]. Han et al. found that during walking, the COP path on the stance foot is rotated 6° inwards, meaning that a diagonal-forward weight transfer occurs during walking [28], which might explain the strong correlation we found in the diagonal-forward direction.

COP distances were negatively correlated with fear of falling, meaning that a participant with better weight shifting capacity will have less fear of falling. However, most associations were low with only two moderate correlations (lateral to the affected side and diagonal-forward to the non-affected side). The etiology of falling is complex and fear of falling is known to be multifactorial with physical, psychological and functional influences [42–44], and this might explain why we generally found lower associations between FES and force platform measures.

Limitations of the study include a limited sample of 36 subjects comprising supervised or independently ambulatory participants. It is acknowledged that this is a selected group of participants but a considerable group among PwS. Mean time post stroke was about three months, and our sample was still attending inpatient rehabilitation, which is common in our setting but probably not outside Europe. Furthermore, we did not assess sensory status of the participants or other clinical characteristics that might affect balance performance. These factors warrant caution when generalizing our results to the whole stroke population.

## Conclusion

In summary, the present study aimed to investigate MWS in lateral and diagonal directions by COP variables measured with a commercially available force platform. PwS showed a significantly smaller MWS towards the affected side and a reduced weight shifting capacity towards the non-affected side in the lateral and diagonal-forward direction compared to HC. PwS also showed a decreased ability to MWS in the diagonal-forward direction towards the affected side in comparison to the non-affected side. Although COP displacements in the AP and L directions are commonly used to assess postural control, we believe that the inclusion of diagonal COP displacements is warranted when evaluating an individual’s limits of stability.

Furthermore, strong correlations in the diagonal-forward direction towards the affected side for balance and gait velocity might imply that treatment should not only focus on practicing weight shifting in an AP and lateral direction but also in the diagonal-forward direction.

With commercially available force platforms, it is now possible to measure MWS in a clinical setting, thus offering clinicians an additional component when addressing balance in PwS. These force platforms often also provide training modalities in order to include this aspect into the rehabilitation programs with the advantage of practicing with an external focus and not having to use the affected arm when reaching.

Future longitudinal and predictive research is needed to investigate if diagonal-forward MWS should be used as part of a standard balance assessment. Evaluating whether integrating specific diagonal MWS in balance training would provide better functional outcome for ambulatory sub-acute patients after stroke seems warranted.

## Supporting information

S1 dataset This is the S1 dataset all collected data. (XLSX)

## References

[1] Feigin VL, Forouzanfar MH, Krishnamurthi R, et al Global and regional burden of stroke during 1990–2010: findings from the Global Burden of Disease Study 2010. Lancet. 2014; 383(9913): 245–254. 24449944 10.1016/s0140-6736(13)61953-4 PMC4181600

[2] Warlow C, Sudlow C, Dennis M, Wardlaw J, Sandercock P. Stroke. Lancet. 2003; 362(9391): 1211–1224. doi: 10.1016/S0140-6736(03)14544-8 14568745 10.1016/S0140-6736(03)14544-8

[3] Tyson SF, Hanley M, Chillala J, Selley A, Tallis RC. Balance disability after stroke. Phys Ther. 2006; 86(1): 30–38. 16386060 10.1093/ptj/86.1.30

[4] Verheyden G, Ruesen C, Gorissen M, et al Postural alignment is altered in people with chronic stroke and related to motor and functional performance. J Neurol Phys Ther. 2014; 38(4): 239–245. doi: 10.1097/NPT.0000000000000054 25198868 10.1097/NPT.0000000000000054

[5] Geurts ACH, de Haart M, van Nes IJW, Duysens J. A review of standing balance recovery from stroke. Gait Posture. 2005; 22(3): 267–281. doi: 10.1016/j.gaitpost.2004.10.002 16214666 10.1016/j.gaitpost.2004.10.002

[6] Langhorne P, Bernhardt J, Kwakkel G. Stroke Care 2, Stroke rehabilitation. Lancet. 2011; 377:1693–1702. 21571152 10.1016/S0140-6736(11)60325-5

[7] Genthon N, Gissot A, Froger J, Rougier P, Perennou D, Posturography in patients with stroke, estimating the percentage of body weight on each foot from a single force platform. Stroke. 2008; 39:489–491. doi: 10.1161/STROKEAHA.107.493478 18174486 10.1161/STROKEAHA.107.493478

[8] Nardone A, Godi M, Grasso M, Guglielmetti S, Schieppati M. Stabilometry is a predictor of gait performance in chronic hemiparetic stroke patients. Gait Posture. 2009; 30(1): 5–10. doi: 10.1016/j.gaitpost.2009.02.006 19318253 10.1016/j.gaitpost.2009.02.006

[9] Mansfield A, Danells CJ, Inness E, Mochizuki G, McIlroy WE. Between-limb synchronization for control of standing balance in individuals with stroke. Clin Biomech. 2011; 26(3): 312–317. 10.1016/j.clinbiomech.2010.10.001 21055854

[10] Shumway–Cook A, Anson D, Halter S. Postural sway biofeedback: its effect on reestablishing stance stability in hemiplegic patients. Arch Phys Med Rehabil. 1988; 69(6): 395–400. 3377664

[11] Weerdesteyn V, de Niet M, van Duijnhoven HJR, Geurts ACH. Falls in individuals with stroke. J Rehabil Res Dev. 2008; 45(8): 1195–1214. 19235120

[12] de Haart M, Geurts A, Huidekoper S, Fasotti L, van Limbeek J. Recovery of standing balance in postacute stroke patients: a rehabilitation cohort study. Arch Phys Med Rehabil. 2004; 85: 886–895. 15179641 10.1016/j.apmr.2003.05.012

[13] Yu E, Abe M, Masani K, Kawashima N, Eto F. Evaluation of postural control in quiet standing using center of mass acceleration: Comparison among the young, the elderly, and people with stroke. Arch Phys Med Rehabil. 2008; 89(6): 1133–1139. doi: 10.1016/j.apmr.2007.10.047 18503811 10.1016/j.apmr.2007.10.047

[14] Karlsson A, Frykberg G. Correlations between force-plate measures for assessment of balance. Clin Biomech. 2000; 15(5): 365–369. 10.1016/s0268-0033(99)00096-0 10758298

[15] Pyöriä O, Era P, Talvitie U. Relationships between standing balance and symmetry measurements in patients following recent strokes (≤ 3 weeks) or older strokes (≥ 6 months). Phys Ther. 2004; 84(2): 128–136. 14744203

[16] Corriveau H, Herbert R, Raiche M, Prince F. Evaluation of postural stability in the elderly with stroke. Arch Phys Med Rehabil. 2004; 85(7): 1095–1101. 15241756 10.1016/j.apmr.2003.09.023

[17] Roerdink M, Geurts ACH, de Haart M, Beek PJ. On the relative contribution of the paretic leg to the control of posture after stroke. Neurorehabil Neural Repair. 2009; 23(3): 267–274. doi: 10.1177/1545968308323928 19074685 10.1177/1545968308323928

[18] Eng JJ, Chu KS. Reliability and comparison of weight-bearing ability during standing tasks for individuals with chronic stroke. Arch Phys Med Rehabil. 2002; 83(8): 1138–1144. 12161837 10.1053/apmr.2002.33644 PMC3501528

[19] de Haart M, Geurts A, Dault M, Nienhuis B, Duysens J. Restoration of weight-shifting capacity in patients with postacute stroke: a rehabilitation cohort study. Arch.Phys. Med. Rehabil. 2005; 86(4):755–762. doi: 10.1016/j.apmr.2004.10.010 15827928 10.1016/j.apmr.2004.10.010

[20] de Oliveira CB, de Medeiros IR, Frota NA, et al Balance control in hemiparetic stroke patients: Main tools for evaluation. J Rehabil Res Dev. 2008; 45(8): 1215–1226. 19235121

[21] Tyson SF, Connell L. How to measure balance in clinical practice. A systematic review of the psychometrics and clinical utility of measures of balance activity for neurological conditions. Clin Rehabil. 2009; 23(9): 824–840. doi: 10.1177/0269215509335018 19656816 10.1177/0269215509335018

[22] Mansfield A, Mochizuki G, Inness EL, McIlroy WE. Clinical correlates of between limb synchronization of standing balance control and fall during inpatient stroke rehabilitation. Neurorehabil Neural Repair. 2012; 26: 627–635. doi: 10.1177/1545968311429688 22275158 10.1177/1545968311429688 PMC4882161

[23] Mizelle C, Rodgers M, Forrester L. Bilateral foot center of pressure measures predict hemiparetic gait velocity. Gait Posture. 2006; 24(3): 356–363. doi: 10.1016/j.gaitpost.2005.11.003 16332441 10.1016/j.gaitpost.2005.11.003

[24] Piirtola M, Era P. Force platform measurements as predictors of falls among older people: a review. Gerontology. 2006; 52(1): 1–16. doi: 10.1159/000089820 16439819 10.1159/000089820

[25] Clark M, Czaja S, Weber R. Older adults and daily living task profiles. Human Factors 1990; 32(5): 537–549. doi: 10.1177/001872089003200504 2074108 10.1177/001872089003200504

[26] Nachreiner N, Findorff M, Wyman J, McCarthy T. Circumstances and consequences of falls in community-dwelling older women. Journal of Women’s Health. 2007; 16(10):1437–1446. doi: 10.1089/jwh.2006.0245 18062759 10.1089/jwh.2006.0245

[27] Chen B, Guo J, Liu M, et al Effect of traditional Chinese exercise on gait and balance for stroke: a systematic review and meta-analysis. PLOS One. 2015; 10(8): e0135932 doi: 10.1371/journal.pone.0135932 26291978 10.1371/journal.pone.0135932 PMC4546302

[28] Han TR, Paik NJ, Im MS. Quantification of the path of center of pressure (COP) using an F-scan in-shoe transducer. Gait Posture. 1999; 10(3): 248–254. 10567757 10.1016/s0966-6362(99)00040-5

[29] Newstead AH, Hinman MR, Tomberlin JA. Reliability of the BBS and balance master limits of stability tests for individuals with brain injury. J Neurol Phys Ther. 2005; 29(1): 18–23. 16386157 10.1097/01.npt.0000282258.74325.cf

[30] Ganesan M, Pal PK, Sendhil KR, Thennarasu K, Usha BR. Quantitative evaluation of balance in patients with spinocerebellar ataxia type 1: a case control study. Parkinsonism Relat Disord. 2009; 15(6): 435–439. doi: 10.1016/j.parkreldis.2008.10.003 19028133 10.1016/j.parkreldis.2008.10.003

[31] Ganesan M, Pasha SA, Pal PK, Yadav R, Gupta A. Direction specific preserved limits of stability in early progressive supranuclear palsy: a dynamic posturographic study. Gait Posture. 2012; 35(4): 625–629. doi: 10.1016/j.gaitpost.2011.12.012 22225854 10.1016/j.gaitpost.2011.12.012

[32] Gray VL, Ivanova TD, Garland SJ. Reliability of center of pressure measures within and between sessions in individuals post-troke and healthy controls. Gait Posture. 2014; 40(1): 198–203. doi: 10.1016/j.gaitpost.2014.03.191 24768116 10.1016/j.gaitpost.2014.03.191

[33] Geronimi M. Reproductibilité intra- et intersessions du test des limites de stabilité sur plateforme podobarométrique. Neurophysiol Clin Neurophysiol. 2014; 44(1):139

[34] Ruhe A, Fejer R, Walker B. The test-retest reliability of centre of pressure measures in bipedal static task conditions—a systematic review of the literature. Gait Posture. 2010; 32(4): 436–445. doi: 10.1016/j.gaitpost.2010.09.012 20947353 10.1016/j.gaitpost.2010.09.012

[35] Knorr S, Brouwer B, Garland SJ. Validity of the Community Balance and Mobility Scale in community-dwelling persons after stroke. Arch Phys Med Rehabil. 2010; 91(6): 890–896. doi: 10.1016/j.apmr.2010.02.010 20510980 10.1016/j.apmr.2010.02.010

[36] Geroin C, Mazzoleni S, Smania N, et al Systematic review of outcome measures of walking training using electromechanical and robotic devices in patients with stroke. J Rehabil Med. 2013; 45(10): 987–996. doi: 10.2340/16501977-1234 24150661 10.2340/16501977-1234

[37] Yardley L, Beyer N, Hauer K, Kempen G, Piot-Ziegler C, Todd C. Development and initial validation of the Falls Efficacy Scale-International (FES-I). Age Ageing. 2005; 34(6): 614–619. doi: 10.1093/ageing/afi196 16267188 10.1093/ageing/afi196

[38] Portney LG, Watkins MP. Foundations of Clinical Research: Applications to Practice. 3^rd^ edition USA: Pearson; 1995.

[39] Goldie PA, Matyas TA, Evans OM, Galea M, Bach TM. Maximum voluntary weight-bearing by the affected and unaffected legs in standing following stroke. Clin Biomech (Bristol, Avon). 1996; 11(6): 333–342. 10.1016/0268-0033(96)00014-9 11415642

[40] Goldie P, Evans O, Matyas T. Performance in the stability limits test during rehabilitation following stroke. Gait Posture. 1996; 4(4): 315–322.

[41] Paillex R, So A. Changes in the standing posture of stroke patients during rehabilitation. Gait Posture. 2005; 21(4): 403–409. doi: 10.1016/j.gaitpost.2004.04.011 15886130 10.1016/j.gaitpost.2004.04.011

[42] Legters K. Fear of falling. Phys Ther. 2002; 82(3): 264–272. 11869155

[43] Hyndman D, Ashburn A, Stack E. Fall events among people with stroke living in the community: circumstances of falls and characteristics of fallers. Arch Phys Med Rehabil. 2002; 83(2): 165–170. 11833018 10.1053/apmr.2002.28030

[44] Hyndman D, Ashburn A. People with stroke living in the community: Attention deficits, balance, ADL ability and falls. Disabil Rehabil. 2003; 25(15): 817–822. doi: 10.1080/0963828031000122221 12851091 10.1080/0963828031000122221

